# Novel Per- and Polyfluoroalkyl Substances Discovered in Cattle Exposed to AFFF-Impacted Groundwater

**DOI:** 10.1021/acs.est.3c03852

**Published:** 2023-08-30

**Authors:** Pradeep Dewapriya, Sandra Nilsson, Sara Ghorbani Gorji, Jake W. O’Brien, Jennifer Bräunig, María José Gómez Ramos, Eric Donaldson, Saer Samanipour, Jonathan W. Martin, Jochen F. Mueller, Sarit L. Kaserzon, Kevin V. Thomas

**Affiliations:** † Queensland Alliance for Environmental Health Sciences (QAEHS), The University of Queensland, 20 Cornwall Street, Woolloongabba 4102 Queensland, Australia; ‡ Department of Chemistry and Physics, University of Almería, Agrifood Campus of International Excellence ceiA3 (ceiA3), Carretera Sacramento s/n, La Cañada de San Urbano, Almería 04120, Spain; § Aviation Medical Specialist, The Australasian Faculty of Occupational & Environmental Medicine (AFOEM), The Royal Australasian College of Physicians (RACP), Sydney, New South Wales 2000, Australia; ∥ Van ‘t Hoff Institute for Molecular Sciences (HIMS), University of Amsterdam, Amsterdam 1090 GD, The Netherlands; ⊥ Department of Environmental Science (ACES, Exposure & Effects), Science for Life Laboratory, Stockholm University, Stockholm 106 91, Sweden

**Keywords:** high-resolution mass spectrometry, non-target analysis, whole blood, PFASs, precursor, biotransformation intermediate, sulfonamides

## Abstract

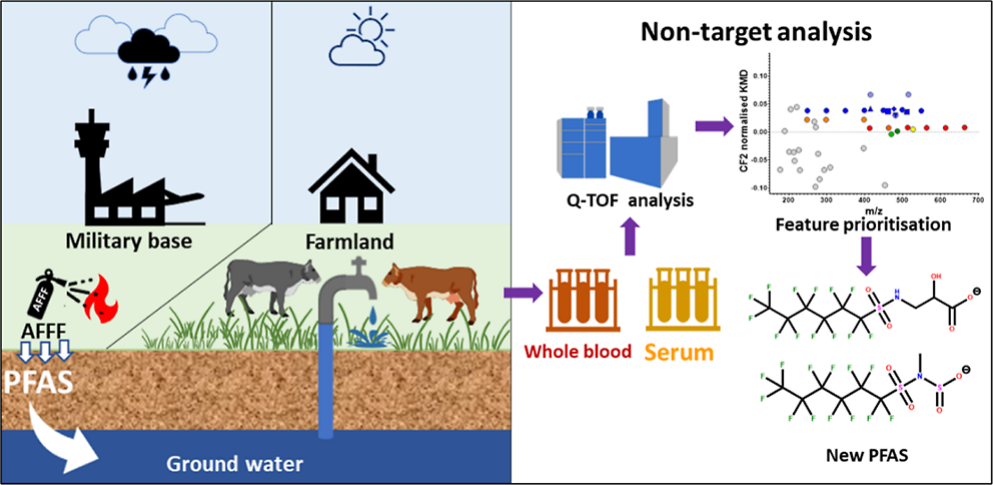

The leaching of per- and polyfluoroalkyl substances (PFASs) from Australian firefighting training grounds has resulted in extensive contamination of groundwater and nearby farmlands. Humans, farm animals, and wildlife in these areas may have been exposed to complex mixtures of PFASs from aqueous film-forming foams (AFFFs). This study aimed to identify PFAS classes in pooled whole blood (*n* = 4) and serum (*n* = 4) from cattle exposed to AFFF-impacted groundwater and potentially discover new PFASs in blood. Thirty PFASs were identified at various levels of confidence (levels 1a–5a), including three novel compounds: (i) perfluorohexanesulfonamido 2-hydroxypropanoic acid (FHxSA-HOPrA), (ii) methyl((perfluorohexyl)sulfonyl)sulfuramidous acid, and (iii) methyl((perfluorooctyl)sulfonyl)sulfuramidous acid, belonging to two different classes. Biotransformation intermediate, perfluorohexanesulfonamido propanoic acid (FHxSA-PrA), hitherto unreported in biological samples, was detected in both whole blood and serum. Furthermore, perfluoroalkyl sulfonamides, including perfluoropropane sulfonamide (FPrSA), perfluorobutane sulfonamide (FBSA), and perfluorohexane sulfonamide (FHxSA) were predominantly detected in whole blood, suggesting that these accumulate in the cell fraction of blood. The suspect screening revealed several fluoroalkyl chain-substituted PFAS. The results suggest that targeting only the major PFASs in the plasma or serum of AFFF-exposed mammals likely underestimates the toxicological risks associated with exposure. Future studies of AFFF-exposed populations should include whole-blood analysis with high-resolution mass spectrometry to understand the true extent of PFAS exposure.

## Abstract 2

This pioneering study utilizes high-resolution mass spectrometry (HRMS) to characterize per- and polyfluoroalkyl substances (PFASs) in contaminated cattle blood and serum, uncovering 30 compounds, including 3 novel PFASs.

## 1 Introduction

Per- and polyfluoroalkyl substances (PFASs) are widely recognized as pervasive environmental contaminants that have adverse ecological and human health impacts.^1,2^ Among their numerous sources, aqueous film-forming foams (AFFFs) used for emergency firefighting and training activities are a major point source of PFAS environmental contamination and human exposure in Australia, and at many contaminated sites globally.^3^ The use of PFAS-containing-AFFF products dates to the late 1960s,^2,4^ but public attention to the environmental fate and impact of its chemical constituents^5^ was not raised until the bioaccumulation properties were identified.^6^ After revealing the global distribution and bioaccumulation potential of perfluorooctane sulfonic acid (PFOS), identification and quantification of PFASs from AFFF-impacted environmental matrices^7^ and biological samples^8^ began to be reported. However, early studies on AFFFs focused on PFOS and perfluorooctanoic acid (PFOA), the major constituents of AFFFs at the time. Nonetheless, early reports such as patents related to PFASs in AFFF formulations indicated a mixture of fluoroalkyl compounds, including higher-molecular-weight precursors to PFOS or PFOA, such as substituted perfluorooctyl sulfonamides and perfluoroheptyl amides.^9^

Generally, PFAS manufacturing processes are known to generate complex PFAS chemistries and a wide range of byproducts.^10−12^ In addition, ∼20% of PFASs in AFFF formulations have the potential to undergo environmental transformation.^13,14^ Therefore, AFFF-contaminated matrices contain a significant amount of unknown organofluorine compounds that are not measurable with the current target analysis.^15^ For example, Koch et al.^16^ showed that 42–58% extractable organofluorine (EOF) content in AFFF-impacted surface water could not be explained by target PFAS analysis. Furthermore, analysis of AFFF-impacted groundwater and biological samples by the total oxidizable precursor (TOP) assay revealed that 25% of the precursors to perfluoroalkyl carboxylates (PFCAs) were unidentifiable.^17^ Thus, it should be kept in mind that target PFAS analysis with today’s available reference standards will only detect a fraction of total PFAS at AFFF-contaminated sites. Therefore, non-target analysis (NTA) and suspect screening approaches, which employ HRMS, have been developed over the last decade as complementary analytical techniques to detect a broader range of PFASs in AFFFs^18−20^ and AFFF-contaminated matrices,^19,21^ including groundwater,^19^ soil,^22^ concrete,^22^ surface water runoff,^23^ and blood serum.^24−26^

Overall, the application of HRMS to identify PFASs in AFFF-exposed individuals remains limited, and relevant information could be gleaned from applying the same approaches to AFFF-exposed farm animals.^27^ Farm animals can be exposed to PFASs from AFFFs at the impacted sites where groundwater is pumped for livestock drinking water. To the best of our knowledge, the application of HRMS analysis to monitor PFAS in farm animals has not been reported previously, even though many studies have reported legacy PFASs in such animals by target analysis.^28−30^ For example, a target analysis of nine PFASs conducted on Holstein cow plasma revealed a higher accumulation of perfluoroalkyl sulfonic acids (PFSAs) in plasma compared to PFCAs.^28^ In another study, 16 PFASs were analyzed and high concentrations of PFOS were reported in cattle serum.^27^ Neither of these former studies reported NTA or suspect screening of PFASs, and the full extent of PFAS contamination in livestock is not well characterized.

The present study optimized a NTA workflow to characterize PFASs in whole blood (hereinafter referred to as “blood”) and serum of cattle that are known to have been exposed to AFFFs or their chemical transformation products in impacted groundwater. The aim was to more comprehensively identify AFFF-derived PFASs that can accumulate in the blood or serum of mammals to inform future exposure and risk assessments at AFFF-contaminated sites and to potentially identify novel PFASs that were not previously reported.

## 2 Materials and Methods

### 2.1 Sample Collection and Pooling

Blood and serum samples from cattle exposed to AFFF-contaminated groundwater were collected (March 2015 to March 2016) from a farm nearby a military establishment in Queensland. Since the late 1970s, PFAS-containing AFFFs, 6% Lightwater produced by 3M, had been used for firefighting and training activities at this facility.^31^ To the best of our knowledge, the composition of the 6% Lightwater used in Australia has not yet been analyzed using HRMS techniques. The historical use, spillage, and leakage from underground storage tanks had been reported; consequently, the groundwater aquifer has been extensively contaminated, including PFOS concentrations in the range of 4.6 ± 4.4 μg/L.^29,32^ A previous study quantified 10 PFAAs in environmental and biological samples, including cattle serum from this site.^29^ The contaminated blood and serum samples used in the current work were collected in 2015–2016 as part of the former investigation^29^ by qualified personnel under the guideline of UQ Ethical Clearance (#ANRFA/ENTOX/153/16). All the samples collected were from a small non-commercial herd (130 cattle including breeders, young cattle, and bulls) held in farmland within the extent of the PFAS groundwater plume. Due to the limited capacity for collecting blood and serum samples, we carefully selected representative cattle from various categories, including breeders, calves, and bulls, for the sample collection process so that a broad representation of the cattle population was captured despite the constraints on sample collection resources and limitations. The blood and serum samples used as the controls were from unused and leftover clinical samples collected (November 2020) from cattle in non-contaminated areas by the School of Veterinary Science, The University of Queensland, Gatton, Queensland. Qualified veterinarians collected these samples (using the same collection protocol and collection tubes) under ethical clearance (ANRFA/QAEHS/421/20). All the samples were collected in appropriate clean sample tubes (BD Vacutainer EDTA and SST II Advance, Plymouth, United Kingdom) and immediately sealed to avoid any contamination. The tubes were transferred to the laboratory on the collection day, where they were frozen (−20 °C) until analysis. Four pooled blood and serum samples from contaminated cattle were prepared by mixing equal volumes (0.3 mL, *n* = 5) from randomly selected individuals. Using the same strategy, four blood and serum pools were also prepared for reference control samples.

### 2.2 Chemicals and Reagents

Native and isotopically labeled PFAS standards were purchased from Wellington Laboratories (Guelph, Ontario, Canada) (Table S3). LC/MS-grade ammonium acetate (99.0%) was from Merck KGaA (Darmstadt, Germany). LC/MS-grade methanol and acetonitrile (ACN) were purchased from Merck Pty Ltd. (Victoria, Australia). Ultrapure laboratory water (18.2 MΩ cm at 25 °C, Milli-Q system, Millipore, Bedford) was used for the sample extraction and chromatographic analysis.

### 2.3 Sample Extraction

Samples from contaminated cattle were thawed from storage at −20 °C and pooled and extracted in January 2021 using previously published methods^24,33^ with slight modifications. The control blood, serum, and fetal bovine serum (FBS) were extracted using the same protocol. Briefly, 1 mL of each of the pooled blood and serum were transferred to 15 mL Eppendorf tubes, spiked with 10 μL (200 μg/L) of mass-labeled internal standards (ISs), and vortexed. Acetonitrile (7.5 mL) was added to the samples to precipitate proteins, and the samples were ultrasonicated for 15 min, followed by centrifugation for 30 min at 5250*g*. The supernatant was filtered using Phenomenex syringe filters (RC membrane 0.2 μm, Lane Cove, Australia) and evaporated to 0.2 mL under a gentle stream of nitrogen. Then, the final volume of the samples was adjusted to 0.5 mL by adding 0.3 mL of Milli-Q water.

### 2.4 UHPLC-QTOF HRMS Analysis

Sample analysis was performed using ultrahigh-performance liquid chromatography (UHPLC, ExionLC AD, AB SCIEX, Ontario, Canada) coupled to a SCIEX X500R quadrupole time-of-flight (QTOF) mass spectrometer (AB SCIEX, Ontario, Canada) operated in electrospray ionization negative mode (ESI^–^). The UHPLC system was equipped with a delay column (Kinetex EVO C18, 100 Å, 5 μm, 2.1 mm × 30 mm; Lane Cove West, NSW, Australia) upstream of the injector to separate instrumental background analytes from sample analytes. The chromatographic separation of analytes was achieved on an ACQUITY UPLC HSS T3 column (100 Å, 1.8 μm, 2.1 mm × 100 mm, Waters, Rydalmere, NSW, Australia) fitted with a VanGuard pre-column (HSS T3, 100 Å, 1.8 μm, 2.1 mm × 5 mm, Waters, Rydalmere, NSW, Australia). The chromatographic flow rate was set at 0.4 mL/min (see Table S1 for mobile phase gradient), and the injection volume was set as 10 μL. The column oven temperature was maintained at 40 °C. Analytes were eluted with a gradient elution program (Table S1) using mobile phase A consisting of Milli-Q water (95%) and methanol (5%) and mobile phase B with methanol (100%). Both mobile phases were fortified with 2 mM ammonium acetate.

For mass spectrometry analysis, two independently optimized data acquisition methods [i.e., SWATH data-independent acquisition and data-dependent analysis (DDA)] were used. High-purity nitrogen was used as the nebulizer, curtain, and collision gases. The full scan mass spectra (MS1, 25,000 mass resolution at *m*/*z* 112.9855) and fragmentation spectra (MS/MS) were recorded using SWATH mode for initial data acquisition. The mass range was set as 100–1200 *m*/*z* for MS1 and 50–1100 *m*/*z* for MS/MS with 12 SWATH windows. The parameters of the SWATH analysis were as follows: ion source temperature, 550 °C; ion spray voltage, −4500 V; curtain gas, 30 L/min; ion source gas 1 and 2, 60 psi; declustering potential, −20 V (DP); and collision energy, −35 V (CE) (Table S2). After feature prioritization, the selected samples were re-injected and mass spectra were recorded in DDA mode to obtain cleaner MS/MS spectra to facilitate the identification of non-target features. MS/MS data were recorded for the top 10 candidate ions above the intensity threshold of 1000 cps for a cycle with dynamic background subtraction. The ion source temperature was set at 600 °C, and the ion spray voltage was −4500 V. Curtain gas and ion source gas 1 and 2 were set as 35 L/min and 70 psi, respectively. The declustering potential was −80 V (DP). The MS/MS data were recorded with multiple collision energies (CEs) ranging from 20 to 60 V.

### 2.5 Quality Assurance and Quality Control

All the relevant quality assurance and quality control (QA/QC) parameters described in our previous work were applied to minimize false-positive and negative identifications.^34^ Briefly, in the laboratory, all the samples were spiked with a suite of mass-labeled internal standards (ISs, Table S3) to monitor instrument conditions throughout the analysis [from sample-to-sample variations, assess potential mass and retention time (RT) drift, and correct the matrix effect, Table S3]. A solvent blank spiked with a mixture of native PFAS reference standards was prepared and injected every 10 samples to monitor instrument performance and any carryover. Procedural blanks (Milli-Q water, ACN, and FBS spiked with IS and extracted), solvent blanks (methanol), and instrument blanks (Milli-Q water) were analyzed alongside samples. Commercially available FBS spiked with isotopically labeled PFAS surrogate standards was used as an additional QA/QC step to monitor the performance of the extraction and data acquisition. System calibration was maintained at less than 2 ppm mass error. Instrument calibration and resolution adjustments were performed automatically using the integrated calibrant delivery system of the SCIEX QTOF system and the SCIEX ESI negative calibration solution (part number 5049910). The mass spectrometer was auto-calibrated at the beginning of each batch and then every 10 injections.

### 2.6 Data Processing and Feature Prioritization

**Figure 1:**
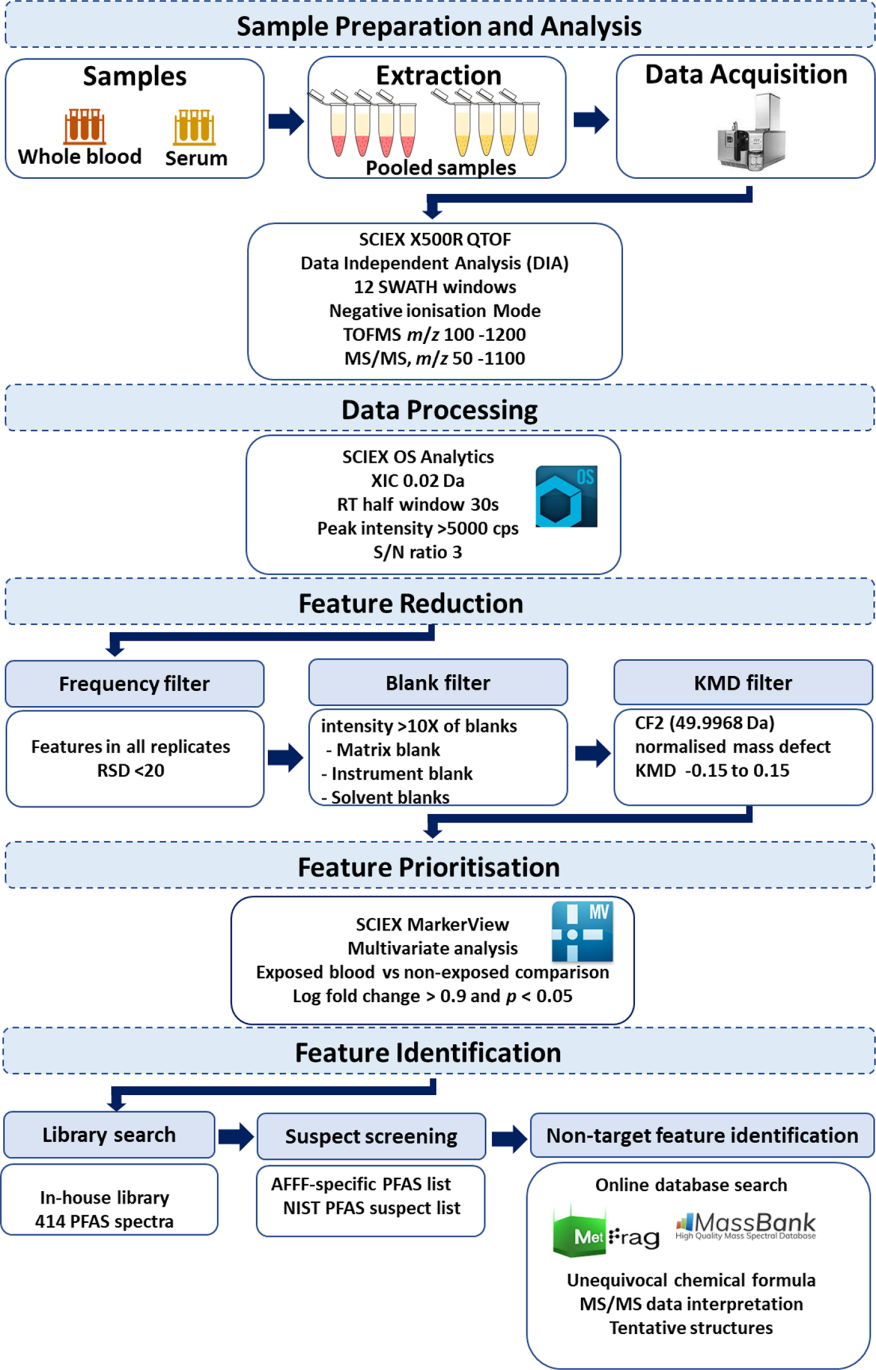
Schematic representation of the workflow. Parameters applied in each step are given in the corresponding frame.

Data processing was performed with SCIEX OS software (version 2.2) using non-target and suspect screening workflows in the analytics module. All the parameters used for creating the feature list are given in Table S4. Briefly, MS1 features from full scan data were extracted with extracted ion chromatogram (XIC) width of 0.02 Da, RT tolerance window of 30 s, and minimum peak width of 3 data points. The minimum peak height was set as 5000 cps, and the signal-to-noise ratio was set to 3. As shown in Figure 1, multiple data filtering layers were applied to identify features by known characteristics of PFASs. Briefly, features in all triplicates with a relative standard deviation (RSD, based on the mean intensity) <20% were considered true, and all the others were excluded from the list. From this list, features with an intensity >10× of the procedural blank sample and eluting at RT between 1 and 13 min (of the 23 min elution; see the Supporting Information for the elution gradient) were selected for further processing. The resulting feature list was subjected to the feature prioritization steps below.

#### 2.6.1 Kendrick Mass Defect Filtering

The CF_2_-normalized Kendrick Mass Defect (KMD) value for all the features was calculated using eqs 1 and 2 below,^35,36^ and features with KMD between −0.15 and 0.15 were selected for further processing
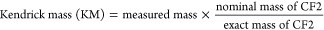
1
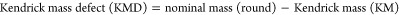
2

#### 2.6.2 Reference Control Filtering

The KMD-filtered feature lists were imported to SCIEX MarkerView software (Version 1.3) for statistical analysis. A *t*-test and principal component analysis were performed to compare the features of the contaminated samples with the reference control samples (i.e., blood and serum samples from non-contaminated cattle). Detailed information about the statistical analysis is provided in the Supporting Information. All the features with log fold change values >0.9, and *p*-value < 0.05, were selected for search against the PFAS library, suspect screening, and non-target feature identification.

### 2.7 Feature Identification

#### 2.7.1 In-House Library Search

The selected features were screened using the SCIEX OS LibraryView tool with an in-house library containing MS/MS spectra for 414 known PFASs acquired under multiple CEs. The analysis was performed with the candidate search algorithm, and the results were sorted by the “Fit” function. The features meeting the following criteria were considered as true library hits: mass error <5 ppm, library match score ≥80, library matches in triplicate, and RT consistent with the molecular mass of the homologues. The library score was generated using the SCIEX OS library score “Fit” function, which calculates a score based on the degree of similarity between the candidate’s MS2 data and an existing library spectrum. The “Fit” score is an indication of the extent to which the library spectrum is encompassed within the unknown spectrum. A score of 100 indicates that all major peaks are present in both the unknown and library spectra.

#### 2.7.2 Suspect Screening

Suspect screening was carried out using SCIEX OS suspect screening tool (target identification module) with an in-house extract ion list consisting of molecular formulas for 1400 AFFF-specific PFASs, as well as the NIST PFAS Suspect List (accessed 2021-03-20).^37^ A hit was considered as true suspect when the mass error was <5 ppm and the % difference of isotope ratio was <5. Finally, MS/MS information was manually assessed, and features with PFAS-specific fragments were selected as true suspects.

#### 2.7.3 Non-target Feature Identification

The remaining features were subjected to non-target feature identification (Figure 1), which consists of three sequential steps: (i) screening the mass of interest (*m*/*z*) together with MS/MS data against MetFragWeb^38^ (PubChem, and two local candidate databases, PubChem_OECDPFAS_largerPFASparts_20220224 and PubChemLite_01Jan2021_exposomics,^39^ were incorporated in the search) and online databases (Massbank Europe; https://massbank.eu/MassBank/Search and MassBank of North America; https://mona.fiehnlab.ucdavis.edu/spectra/search) to identify candidate molecules, (ii) confirming the elemental composition of the candidates and use of isotope patterns, as well as rings and double bonds (RDBs) to reduce the list of candidates, and (iii) manual interpretation of MS/MS data to identify the structure or to elucidate a tentative structure.

Feature identification confidence levels for all the above steps were assigned based on a recently introduced PFAS identification confidence scale, which ranges from level 1a (confirmed by reference standard) to level 5 (exact masses of interest) (see the Supporting Information for more information on the definition of the identification confidence levels).^40^

## 3 Results and Discussion

### 3.1 Non-target Feature Finding

Based on the initial aligned feature list from the HRMS analysis of blood and sera, a total of 11,065 and 8717 features were extracted, respectively. To efficiently detect potential PFAS from this extensive list of features, it was essential to implement comprehensive, yet cost- and time-effective feature prioritization strategies (Figure 1). One such method is the use of mass defect as a preliminary feature filtering technique to extract potential PFAS features.^35^ Generally, PFASs exhibit low or negative mass defects due to the replacement of hydrogen (1.0078 Da) atoms in the carbon backbone with fluorine (18.9984 Da). This unique characteristic has been effectively used in workflows to discover novel PFAS in several previous NTA studies.^19,23,41^ The use of a CF2-normalized mass defect plot (KMD vs *m*/*z*) further facilitates the visualization of prospective PFAS homologues that differ by −CF2– units in complex HRMS feature lists.^42^ However, KMD filtering alone was unable to clearly reveal homologous series in these samples, likely due to the complexity of the data acquired from biological samples. Nevertheless, a combination of the reference control filtering with multivariate analysis proved to be effective at reducing the number of potential PFAS features in blood and serum to 261 and 480, respectively (with some features present in both blood and serum samples). These features were strongly associated (*p*-value < 0.05) with contaminated cattle blood and serum, similar to Rotander et al. where the case-control filtering strategy was highly efficient for filtering PFASs from complex serum feature lists.^25^

### 3.2 In-House MS/MS Library Search

Fifteen features were confidently annotated as PFASs using the in-house fluorochemical library (SCIEX 2.0) screening (Table 1), and 12 of these (PFPrS, PFBS, PFPeS, FHxSA, PFHxS, PFOA, PFHpS, PFNA, PFOS, PFDA, PFNS, and PFUnDa) were confirmed to level 1a (i.e., confirmed by reference standard). Additionally, Cl-PFOS (C_8_HClF_16_O_3_S) was identified to be level 1b (i.e., indistinguishable from reference standard based on MS/MS fragmentation). For FPrSA (C_3_H_2_F_7_NO_2_S) and FBSA (C_4_H_2_F_9_NO_2_S), confidence level 2a (i.e., probable by diagnostic fragmentation evidence) was assigned as reference standards for these two compounds are not available currently in hand.^43^ Notably, certain compounds identified in the previous target analysis (PFBA, PFPeA, PFHxA, and PFHpA) were not detectable in the current study. In the previous analysis, these compounds were found to be present in individual cow serum at concentrations of approximately 0.55, <0.5, <0.5, and <0.1 μg/L, respectively.^29^ Failing to detect these compounds in the current work may be attributed to their lower concentrations in the pooled samples. This implies that the concentrations of the compounds we did detect in this work may be higher compared to some of those included in the target analysis. Therefore, combining target analysis, HRMS-based suspect screening, and NTA is necessary to characterize PFAS exposure comprehensively.

**Table 1:**
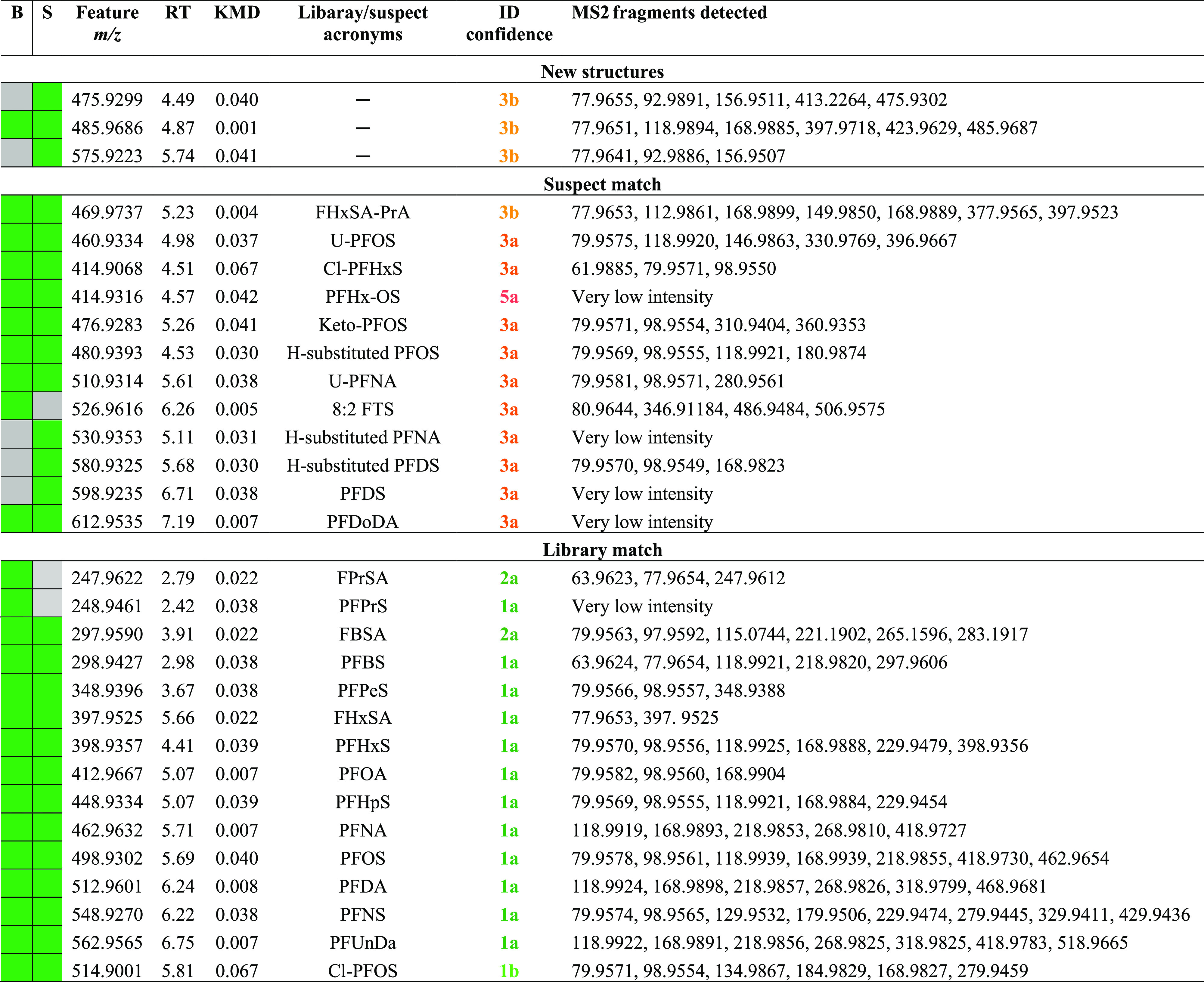
Features Prioritized for Identification^a^

a Mass-to-charge ratio (*m/z*), RT, KMD, identification (ID) confidence,^40^ and the corresponding MS/MS (MS2) fragments are given for each feature. Green squares indicate the presence of the feature in whole blood (B) and serum (S) samples.

Nevertheless, three shorter-chain perfluoroalkane sulfonamides (FPrSA, FBSA, and FHxSA) and two shorter-chain perfluorosulfonic acids (PFPrS and PFPeS) were identified. However, the relative intensities of these shorter-chain PFASs were substantially lower compared to PFOS and PFHxS (Figure S1). Notably, shorter-chain sulfonamides were predominantly detected in blood relative to sera, suggesting that these substances have relatively high affinities toward the cell component of blood. Similar observations have been reported from whole-blood analysis of humans and animals before.^11^ For example, Kärrman et al.^44^ reported high levels of perfluorooctanesulfonamide (FOSA) in human blood compared to plasma. A study by Poothong et al. found the highest concentration of FOSA in blood relative to plasma and serum.^45^ Such observations are unusual for most other PFAS that are routinely quantified in targeted analysis, whereby the highest concentrations are found in serum or plasma, in part due to their affinity for proteins, including albumin. It is noteworthy that only a few studies previously reported shorter-chain (<C6) sulfonamides in human^26^ and animal samples,^46^ likely due to a majority of studies conducted with serum or plasma. Our data suggest that future biomonitoring studies of PFASs at AFFF-contaminated sites should consider analyzing blood, or both blood and serum, to determine the true extent of PFAS exposure.

The shorter-chain (<C6) and so-called ultra-short-chain (<C3) PFASs have rarely been considered in biomonitoring due to their lower bioaccumulative potential. Nonetheless, short-chain and ultra-short-chain PFASs have been detected in several studies of human or biological samples.^26,46,47^ The C4 perfluorosulfonamide identified in cattle here, FBSA, can induce abnormal behaviors and disrupt normal gene expression in embryonic zebrafish;^48^ thus, short-chain and ultra-short-chain PFASs should be considered in future monitoring studies to more fully understand the health implications of AFFF exposure.

### 3.3 Suspect Screening

Eleven more PFASs (Table 1), in addition to the ones discovered with the above library search, were detected by suspect screening. Notably, suspect screening revealed the presence of U-PFOS, U-PFNA, keto-PFOS, and H-substituted PFOS, PFNA, and PFDS. It is important to note that a preliminary suspect match to the monoisotopic mass alone was not sufficient for confidently identifying a feature as a PFAS, and a thorough interpretation of MS/MS data was necessary to confirm the identity of the features that met the quality criteria. This was exemplified in our data with the identification of U-PFOS, which required careful analysis and is described in further detail in the Supporting Information (Figures S2–S5). Except for one feature (i.e., *m*/*z* 414.9315), for identification of all the other hydrogen-substituted PFASs, confidence level 3a (positional isomer candidates) was assigned.^40^ The feature with accurate mass *m*/*z* 414.9315 (C_6_HF_13_SO_4_) returned matches for two structural isomers: either oxygen-substituted PFHpS (O-PFHpS) or perfluorohexane sulfate (PFHx-OS). Due to its low intensity and correspondingly poor MS/MS spectra, it was not possible to clearly elucidate the isomer structure; hence, level 5b confidence (non-target PFAS exact mass of interest) was assigned. Previously, Rotander et al.^25^ reported O-PFHpS in firefighter serum samples by interpreting the MS/MS data. McDonough et al.^26^ also reported the same feature in serum but could not distinguish the isomers due to low peak area. Several previous studies have shown the frequent detection of substituted PFAS from AFFF-impacted sites^19^ and human serum,^26^ suggestive of these compounds’ biopersistence. Furthermore, it has been shown that U-PFOS accumulated in mice dosed with AFFF.^49^ Despite their apparent biopersistence, these PFAS have not yet been included in PFAS exposure monitoring as the reference standards are not currently available commercially.

### 3.4 Non-target PFAS

**Figure 2:**
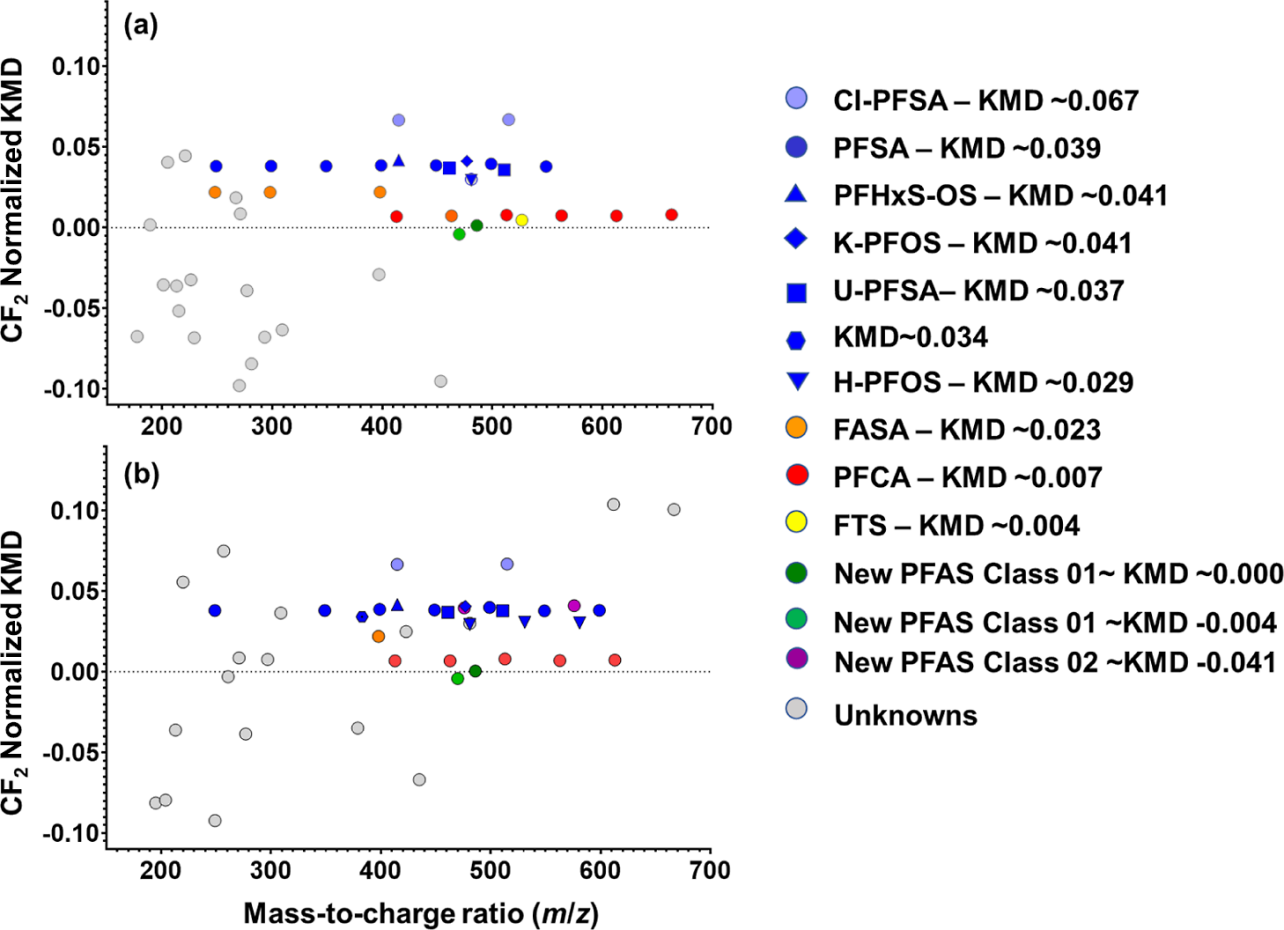
KMD plots of the prioritized features from whole blood (a) and sera (b) with masses (MS1) normalized to CF_2_. Colored markers are those that are showing the identified fragments, and the same color markers represent homologues.

The CF2-normalized KMD plots (Figure 2) were then used to visualize all the candidate features, including 45 features from blood and 44 features from serum. These plots were instrumental in confirming the PFASs identified from library search and suspect screening. Four distinct homologous series, namely, the PFCAs, PFSAs, perfluoroalkane sulfonamides (FASA), and Cl-PFSA, were prominently observed in the KMD plots. The identification of suspected PFAS features and substituted PFAS was relatively straightforward as these compounds were homologous and thus horizontally adjacent to each other in these plots. For the remaining features in the KMD plots, we performed non-target feature identification (Figure 1). Using experimental MS/MS data analysis, we elucidated the structures of two different PFAS classes that have not been reported in any environmental or biological matrix before.

**Figure 3:**
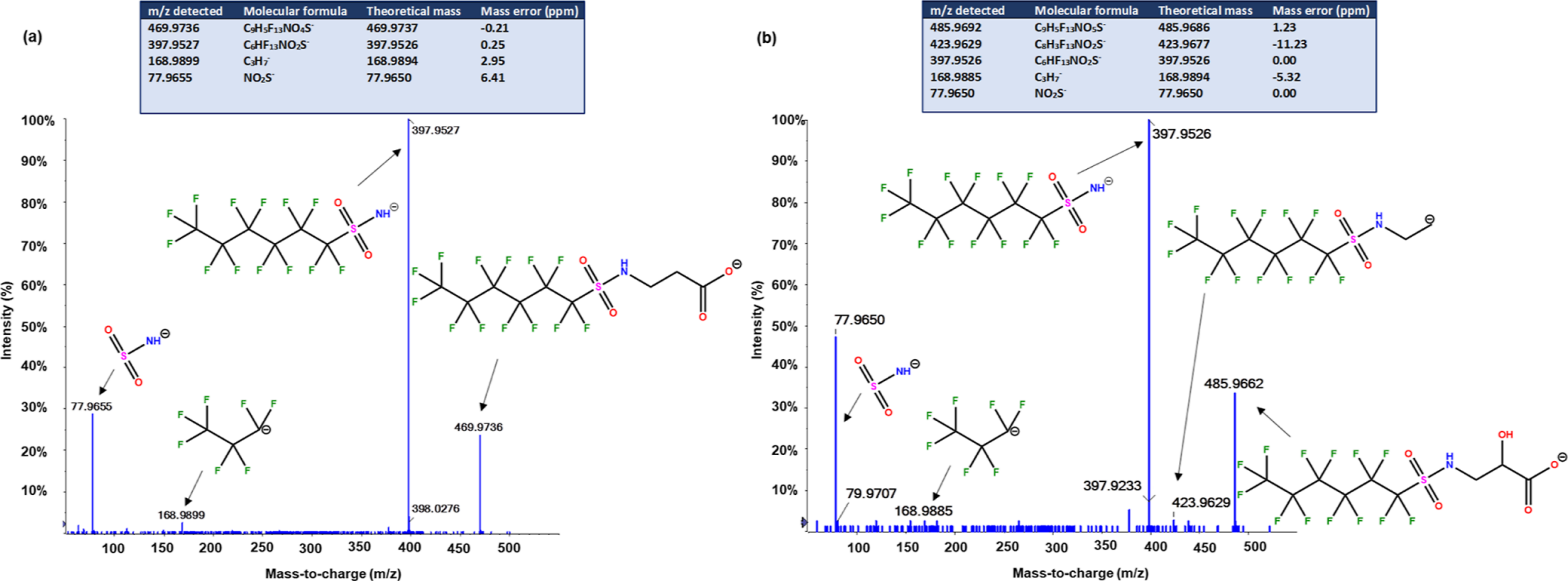
MS/MS spectrum of (a) *m/z* 469.9736 and (b) *m/z* 485.9662 acquired in DDA mode. The inset table shows the fragmentation information, corresponding molecular formula generated, and mass error for each fragment identified. Proposed structures for the major identified fragments are shown next to each fragment. Collision energy (CE) = 35 ± 15 eV.

Two features (*m*/*z* 469.9736 and 485.9662) with comparable MS/MS spectra were abundantly detected in blood. Their elemental compositions were proposed as C_9_H_5_F_13_NO_4_S^–^ and C_9_H_5_F_13_NO_5_S^–^, indicating that *m*/*z* 485.9662 was an oxygenated analogue of *m*/*z* 469.9736. MetFrag search for *m*/*z* 469.9736 returned perfluorohexanesulfonamido propanoic acid (FHxSA-PrA) as a candidate with a high MS/MS similarity score (3 of 3), whereas no hits were found for *m*/*z* 485.9662. FHxSA-PrA is a structural isomer of *N*-methylperfluorohexane sulfonamido acetic acid (MeFHxSAA), which has been detected in AFFF and many environmental matrices such as soil, groundwater, and drinking water.^19,50^ A closer inspection of the MS/MS fragmentation pattern and comparison with the MS/MS data reported in the literature^19^ revealed that the feature detected was not MeFHxSAA but rather a structurally different isomer. More specifically, the fragment ions *m*/*z* 77.9655 (SO_3_^–^) and 397.9527 (C_6_F_13_SO_3_NH) suggested a perfluoroalkyl sulfonamide as a base structure (Figure 3), and this fragmentation pattern was consistent with an established PFAS class (*N*-sulfopropylperfluoroalkane sulfonamide) reported by Barzen-Hanson et al.^19^ Furthermore, a net neutral loss of 72.0211 Da (C_3_H_4_O_2_) suggested a propionate group attached to the base structure, indicating the structure as FHxSA-PrA. The closely related feature at *m*/*z* 485.9662 shared the same base fluorohexyl sulfonamide structure, indicating that the additional oxygen atom could be a hydroxyl group on the propyl chain. A minor fragment ion observed at *m*/*z* 423.9629 (C_8_H_3_F_13_NO_2_S^–^) suggested that the hydroxyl group was next to the terminal acid functionality, and the 2-hydroxy propionate is attached to the sulfonamide head group. Based on this, the structure was proposed (level 3b, fragmentation-based candidates) as perfluorohexanesulfonamido 2-hydroxypropanoic acid (FHxSA-HOPrA), which has not been reported previously.

FASA-PrA are considered transformation intermediates of zwitterionic PFASs that are abundant in AFFFs.^14,51^*N*-dimethyl aminopropyl perfluorohexane sulfonamide (AmPr-FHxSA)^52^ and *N*-trimethylammoniopropyl perfluorooctane sulfonamide (TAmPr-FOSA)^21^ are two examples that have been studied to understand the abiotic and biotic transformation pathways leading to FASA-PrA. Soil microbes enriched with methane and acetate possess a capacity to transform tertiary ammonium AmPr-FHxSA to FHxSA-PrA as an intermediate that eventually degrades to FHxSA.^21,52^ To the best of our knowledge, FASA-PrAs have not been detected in any environmental samples, including blood and serum. These compounds may not have been detected before because many studies focus only on a selected set of targeted PFAS. Our findings need confirmation from further investigations, including an assessment of biotransformation and bioaccumulation of this ECF-based polyfluoroalkyl substance in blood and serum.

**Figure 4:**
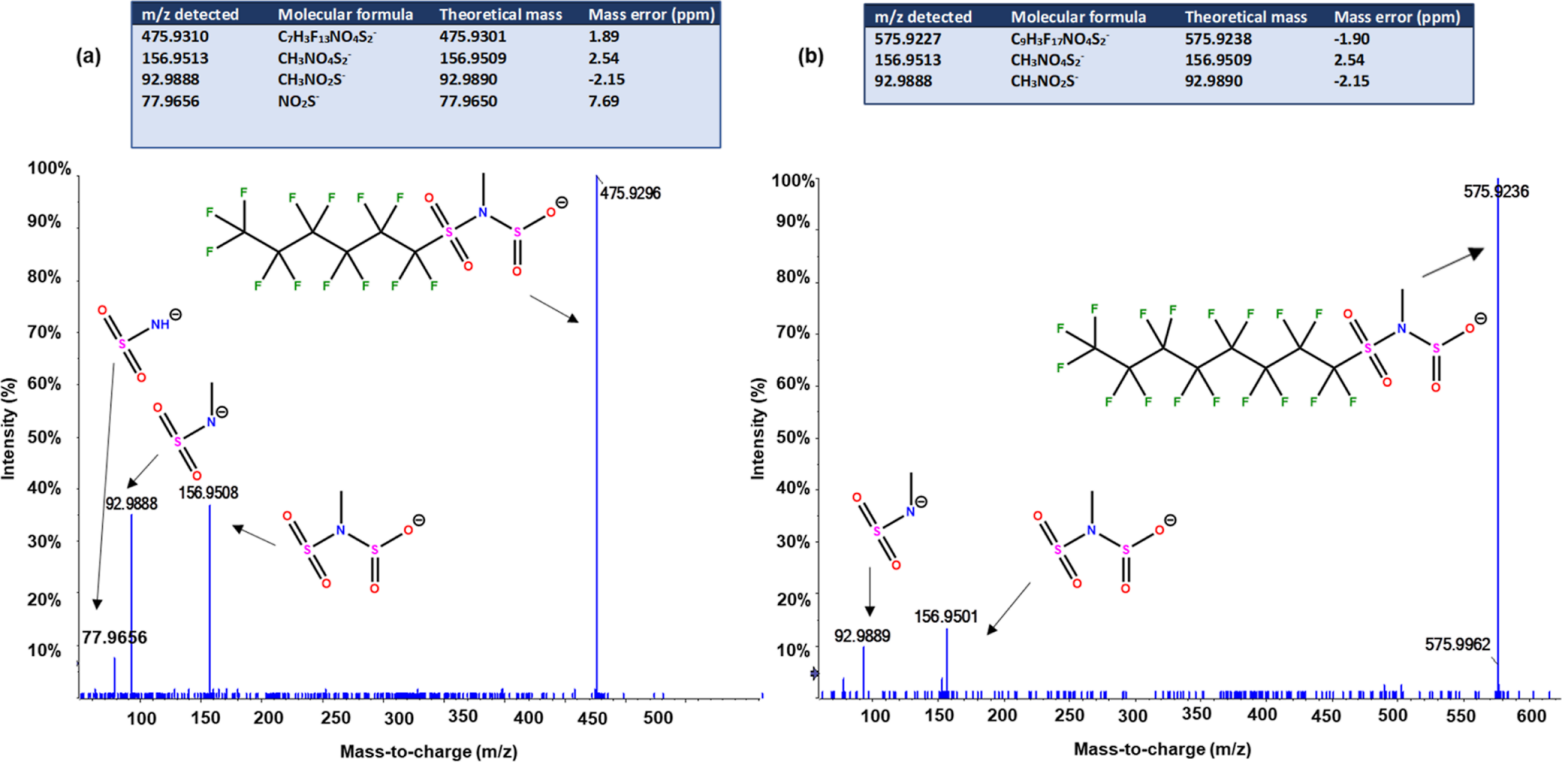
MS/MS spectra of (a) *m/z* 475.9310 and (b) *m/z* 575.9227 acquired in DDA mode. The inset table shows the fragmentation information, corresponding molecular formula generated, and mass error for each fragment identified. Proposed structures for the identified major fragments are shown next to each fragment. Collision energy (CE) = 35 ± 15 eV.

The second class of PFASs was also abundantly detected in the serum samples (Table 1, Figure 2). Two homologous molecules, *m*/*z* 475.9310 (C_7_H_3_F_13_NO_4_S_2_^–^) and *m*/*z* 575.9227 (C_9_H_3_F_17_NO_4_S_2_^–^), which differed by a C_2_F_4_ unit (i.e., 99.9917) provided further evidence for fluorinated molecules. However, no literature matches were found for these masses, suggesting that these two homologues could be unidentified PFASs. Despite being adjacent to the PFSA homologues series in the KMD plot (Figure 2), the absence of a characteristic sulfonate fragment ion (SO_3_^–^, *m*/*z* 79.9574) in the MS/MS spectra indicated that these two molecules belonged to a distinct PFAS class. Instead, spectra showed fragment ions corresponding to NO_2_S^–^ and CH_3_NO_2_^–^ (*m*/*z* 77.9656 and 92.9880, respectively), which were indicative of a class based on an *N*-methyl sulfonamide head group. The neutral losses correspond to C_6_F_13_ and C_8_F_17_, respectively (i.e., 318.97 and 418.97 Da), indicating that C_6_ and C_8_ perfluoroalkyl chains are bound to the sulfonamide head group. A 63.9625 Da mass difference between two adjacent fragment ions at *m*/*z* 92.9888 and *m*/*z* 156.9513 suggested the presence of additional SO_2_^–^ functionality bound to the *N*-methyl sulfonamide. Based on these data, the structures of the molecules were proposed, as shown in Figure 4.

### 3.5 Unknowns

A total of 34 prioritized features remained unidentified due to a lack of evidence to elucidate the structures confidently. The database searches with monoisotopic mass and fragment information did not return any convincing hits for these features. The available MS/MS information was insufficient to draw a structural conclusion. All the unknown features, RT, KMD value, and MS/MS information are shown in the Supporting Information (Table S5). The lists of the unknown together with known PFASs have been submitted to the Zenodo repository (10.5281/zenodo.7905643). This may be helpful to others for future identification of PFASs from AFFF-contaminated samples.

### 3.6 Environmental Implications and Limitations

The results of the analysis revealed the presence of a range of PFASs, including three novel compounds that had not been previously reported from any AFFF-impacted matrices. The detection of these compounds highlighted the power and importance of non-target and suspect screening in PFAS biomonitoring. The three new compounds identified in this work are structurally similar to the classes of PFASs that are generally considered precursors and are known to readily biotransform into stable PFAAs. The detection of these compounds in the blood and serum of animals exposed to contaminated groundwater warrants further research on the biopersistent nature of these compounds. Due to the lack of available toxicological data for many known PFASs, it is challenging to assess the potential health risks associated with these compounds. Our data (Figure S1) revealed that the intensity of these new compounds exceeded that of certain known PFASs currently monitored by target analysis. Consequently, we recognized the importance of quantifying or semi-quantifying these new compounds in future research to facilitate toxicological studies.

It should be noted that the study focused on negative ionization, which means that PFASs that may only ionize in positive mode (i.e., cationic and zwitterionic) were essentially missed. Given that AFFFs contain numerous cationic and zwitterionic PFASs, developing HRMS methods capable of detecting compounds ionized in positive mode would be crucial for obtaining valuable information in future exposure monitoring efforts. While the current study focuses solely on NTA of contaminated blood and serum, conducting a comprehensive analysis of contaminated soil and groundwater from the same site, along with the blood and serum samples, could yield more valuable information. This additional data could include insights into the PFAS chemistry of AFFF used in the area, source tracking, identification of transformation intermediates, and assessment of the environmental/biopersistence of PFAS compounds not monitored in target analysis. All three novel compounds, as well as many other PFASs identified in this study, are ECF-based PFASs, which are the major constituents of 3M-manufactured AFFF formulations. Before introducing fluorotelomer-based AFFFs in Australia in 2005, for nearly 25 years, 3M Lightwater AFFFs (both the 6 and 3% concentrates) had been extensively used for various firefighting and training activities.^32^ Our data further emphasize that AFFF formulations are complex mixtures of PFASs, and more research is needed to understand the chemistry, environmental fate, and environmental burden of AFFF-derived PFASs.

## Supporting Information Available

Details of statistical analysis, definitions of confidence levels, detailed information on confirming the presence of U-PFOS, library spectral match of PFEtCHxS, SWATH-MS/MS data of mass of interest (*m*/*z* 460.9334), DDA MS/MS spectrum of PFEtCHxS reference standards, DDA MS/MS spectrum of *m*/*z* 460.9334 in the sample, chromatographic gradient information, SWATH data acquisition parameters, details of the mass-labeled standards, non-target feature extraction parameters, relative intensity of PFAS identified through NTA and suspect screening, and details of the features that remained unidentified (PDF)
