# Supplementary material for: Novel Per- and Polyfluoroalkyl Substances Discovered in Cattle Exposed to AFFF-Impacted Groundwater
Source: Environ Sci Technol. 2023 Aug 30;57(36):13635–45. doi: 10.1021/acs.est.3c03852 (PMC10501377; doi:10.1021/acs.est.3c03852)
Supplement: Supplementary file 1 — es3c03852_si_001.pdf [file es3c03852_si_001.pdf]

Supporting Information

Novel Per- and Polyfluoroalkyl Substances Discovered in Cattle Exposed to AFFF-Impacted Groundwater

Pradeep Dewapriya[a]*, Sandra Nilsson[a], Sara Ghorbani Gorji[a], Jake W. O'Brien[a,d], Jennifer Bräunig[a], María JoséGómez Ramos[b], Eric Donaldson[c], Saer Samanipour[a,d], Jonathan W. Martin[e], Jochen F. Mueller[a], Sarit L. Kaserzon[a], Kevin V. Thomas[a]

[a] Queensland Alliance for Environmental Health Sciences (QAEHS), The University of Queensland, 20 Cornwall Street, Woolloongabba 4102, QLD, Australia.

[b] Department of Chemistry and Physics, University of Almería, Agrifood Campus of International Excellence ceiA3 (ceiA3), Carretera Sacramento s/n, La Cañada de San Urbano, 04120 Almería, Spain.

[c] Aviation Medical Specialist, The Australasian Faculty of Occupational & Environmental Medicine (AFOEM), The Royal Australasian College of Physicians (RACP), Sydney NSW 2000, Australia.

[d] Van 't Hoff Institute for Molecular Sciences (HIMS), University of Amsterdam, 1090 GD Amsterdam, The Netherlands.

[e] Department of Environmental Science (ACES, Exposure & Effects), Science for Life Laboratory, Stockholm University, Stockholm 106 91, Sweden.

*Corresponding author: p.dewapriya@uq.edu.au

Number of Pages: 12

Number of Figures: 5

Number of Tables: 5

Number of References: 1

Statistical analysis

Statistical data analysis was conducted using MarkerView software (Version 1.3). A peak list, consisting of the mass of interest and intensities in respective samples, was created from the features that were prioritized from the original feature list extracted using SCIEX OS. The peak list was then imported into MarkerView as a generic text file. Prior to performing principal component analysis (PCA), the peak responses were scaled using the pareto-scaling method. This method involved subtracting each value by the average and dividing it by the square root of the standard deviation. This prevented intense peaks from dominating the PCA analysis while also giving more importance to peaks with good signal-to-noise ratios. The analysis was performed with normalization and without normalization of the data using the area of ^{13}C-PFOS and total area sums. However, normalization of the data did not affect the outcome of the results. In both cases, clear separation of control and exposed blood and sera was observed.

To identify significantly different variables between the control and exposed groups, a t-test was performed using the integrated function of the MarkerView software. The t-test was applied to every variable in the peak table and determined whether the mean for each group was significantly different given the standard deviation and the number of samples. The software automatically compared all groups pairwise and each group to all the others. The results of the t-test indicated how well each variable (mass) distinguished the control and exposed groups that had been assigned to each sample before the analysis. The software reported the results as a p-value, which represented the probability that the observed difference occurred by chance. Variables that had a low p-value (<0.05) and a large fold change (>0.9) were the most distinguishable features between the two groups. These variables were selected for further processing.

Assigning the confidence level for identified features

Identification confidence for each feature prioritised was given based on the confidence scale recently introduced by Charbonnet et. al. [1] Below are the complete definitions for the different identification levels. The details were extracted from the original publication.[1]

Level	Identification Confidence	Description
**Level 1a**	Confirmed by reference Standards	Confirmed by matching a feature's exact mass, isotope pattern, retention time, and MS/MS spectrum to those of an analytical reference standard.
**Level 1b**	Indistinguishable from Reference Standard	Confirmed with an analytical reference standard despite the existence of known structures with potentially indistinguishable fragments.
**Level 2a**	Probable by Library Match	Identified by accurate mass, mass defect, and isotopic pattern and matching an MS/MS spectrum in a mass spectral library
**Level 2b**	Probable by Diagnostic Fragmentation Evidence	Confirmed with the other forms of diagnostic evidence in addition to MS/MS fragmentation (e.g., parent compound identity, ionization behaviour, synthesis pathway).
**Level 2c**	Probable by Diagnostic Homologue Evidence	At least two other homologues are identified with level 2a or greater certainty, when the spectra data are insufficient.
**Level 3a**	Positional Isomer Candidates	The presence of a particular functional group is confirmed by fragmentation, but the position of the functional group is ambiguous.
**Level 3b**	Fragmentation-Based Candidates	The candidate structures informed by their MS/MS spectra.
**Level 3c**	Circumstantial Candidates by Fragmentation Evidence	Identified by the circumstantial evidence that may derive from knowledge of the experimental design and/or subclass of PFASs investigated.
**Level 3d**	Circumstantial Candidates by Homologue	Identified by distinct patterns of homologues with sufficient circumstantial evidence.

	Evidence	
**Level 4**	Unequivocal Molecular Formula	The mass and isotopic pattern of the analyte must allow the unequivocal determination of a molecular formula.
**Level 5a**	PFAS Suspect Screening Exact Mass Match	Suspect match to a feature with the same exact mass as feature on a suspect list.
**Level 5b**	Nontarget PFAS Exact Mass of Interest	A filtered feature have an elevated likelihood of being novel PFAS.

Confirming the presence of U-PFOS in the sample

A feature with *m/z* 460.9334 detected in all the whole blood and serum samples returned a match to a closely eluting analytical standard perfluoro-4-ethyl cyclohexane (PFEtCHxS, $C_8F_{15}O_3S^-$, Figure S1). However, MS/MS fragmentation was inconsistent with the standard and the spectra reported in the literature. The major fragment ions of PFEtCHxS (*m/z* 98.9556 and 380.9759) were not detected in the samples indicating that the feature could be a different isomer of the same chemical formula. The SWATH MS/MS data was insufficient to draw a clear conclusion on the detected feature (Figure S2). A few selected samples were reanalysed to confirm the detected peak using information-dependent analysis (IDA) with different collision energies (-20, -35, -45, and -65 eV). The IDA MS/MS spectrum (Figure S5) showed fragments corresponding to fluoroalkenes $C_4F_7^-$ (*m/z* 280.9835) and $C_3F_5^-$ (*m/z* 230.9787) confirming that the detected feature is unsaturated-PFOS (U-PFOS).

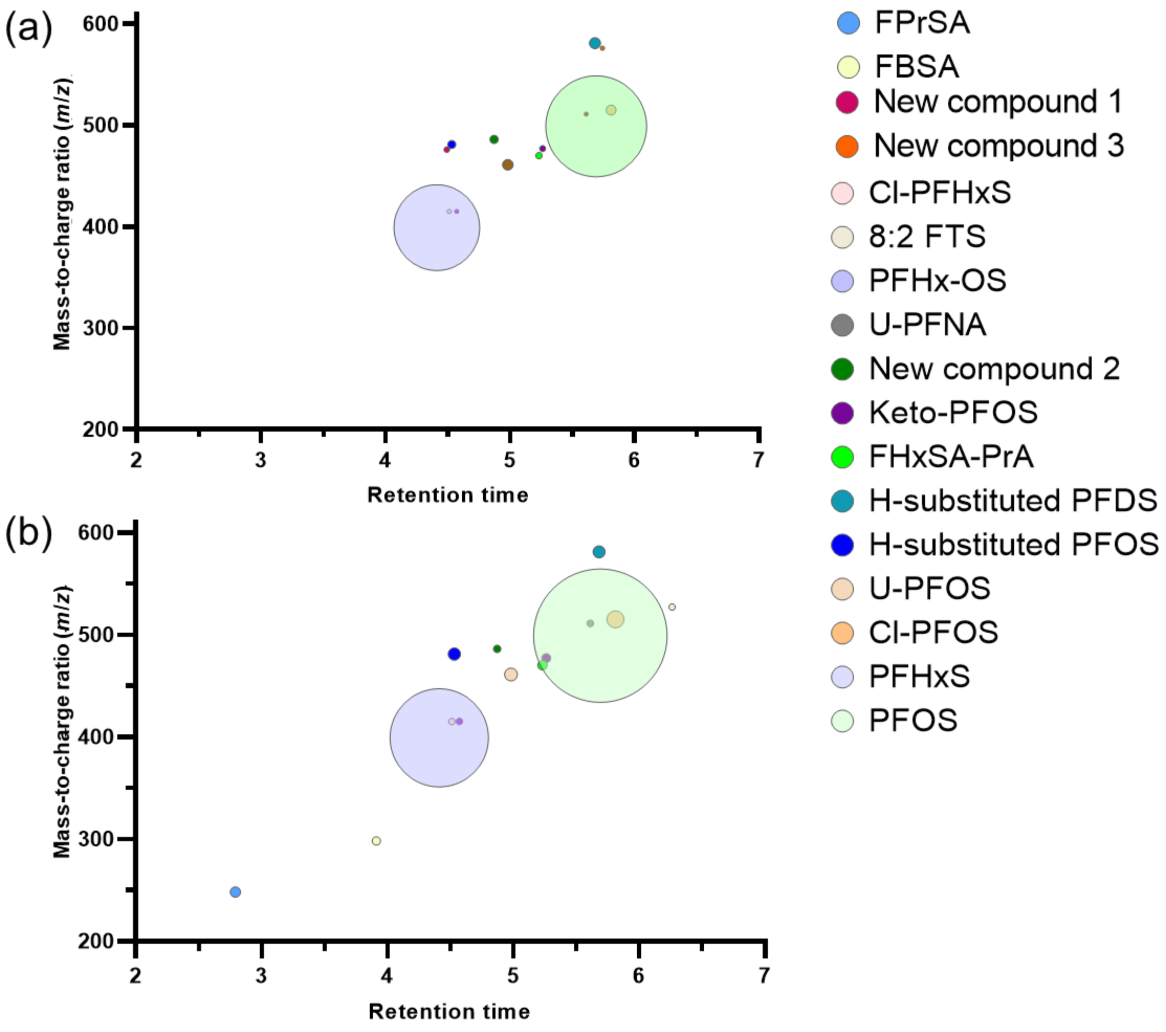

Figure S1: Comparison of PFAS intensities identified using non-target analysis and suspect screening in cow whole blood (a) and serum (b) where the size of the circle shows the intensity. All the intensities are normalised to PFOS internal standard intensity.

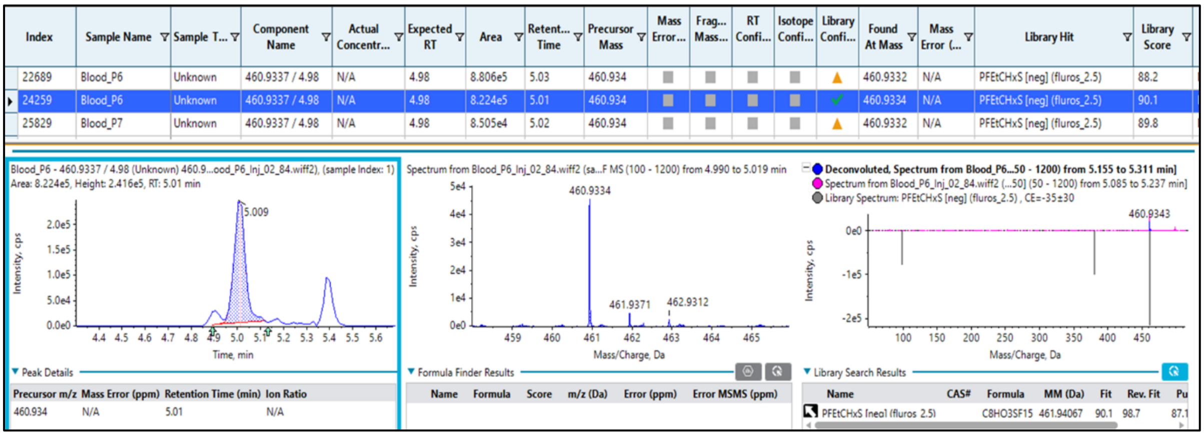

Figure S2. Mass of interest *m*/*z* 460.93 showing the suspect matches to PFEtCHxS.

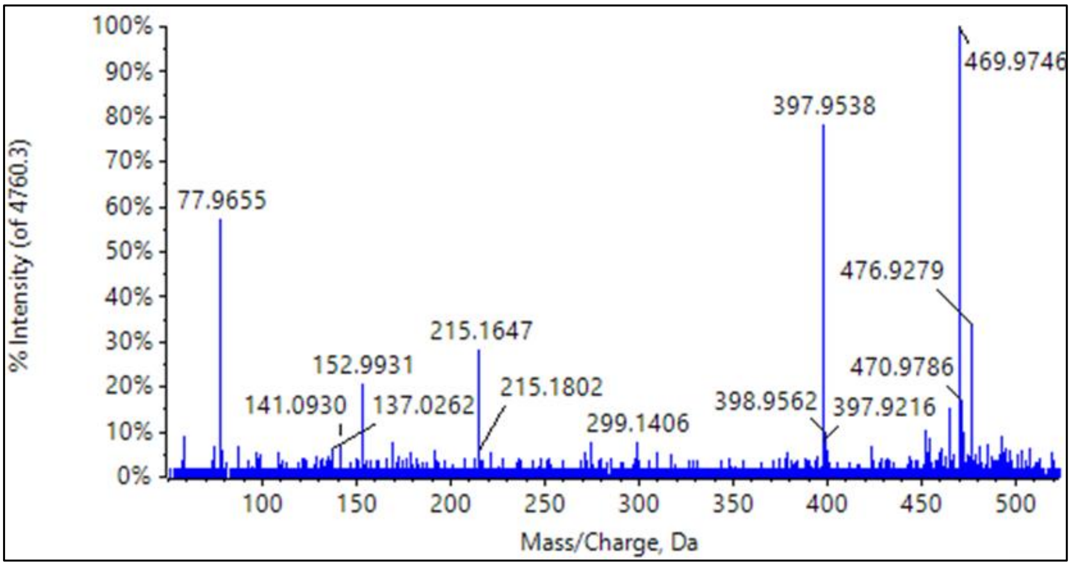

Figure S3. SWATH MS/MS spectrum of mass of interest *m*/*z* 460.9334 from the sample.

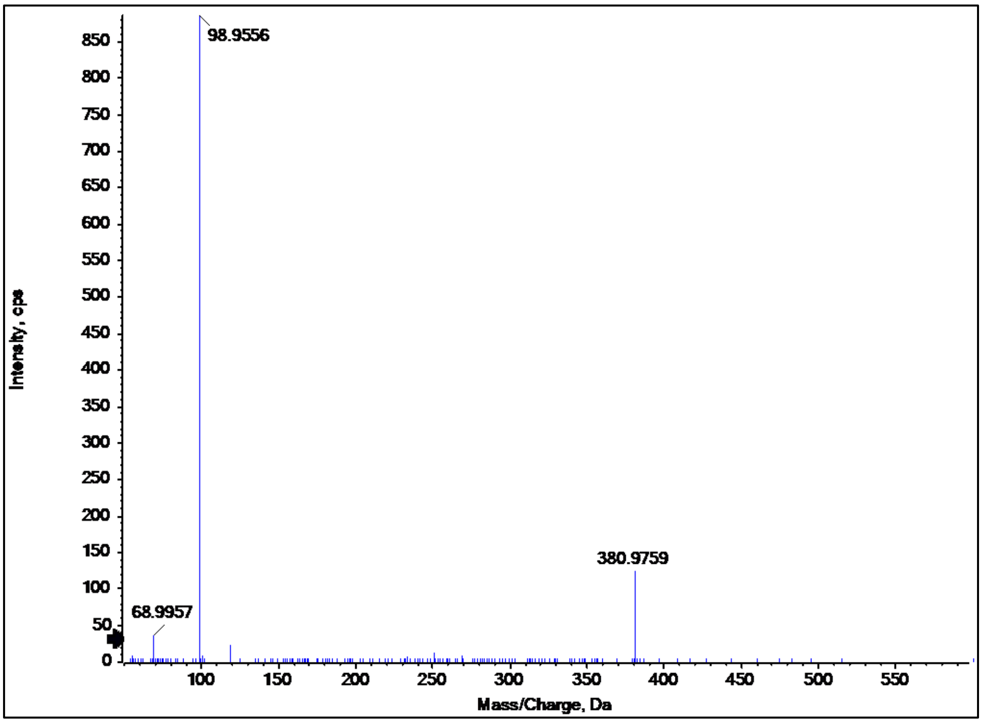

Figure S4. MS/MS spectrum of mass of interest *m*/*z* 460.9334 in the reference standard acquired using DDA mode (CE -60 eV).

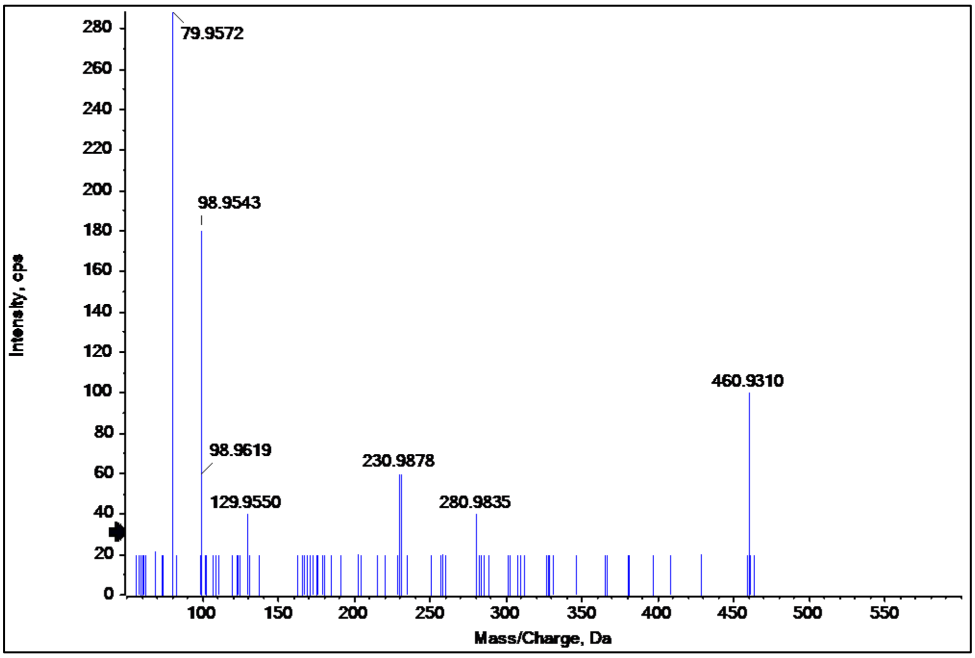

Figure S5. MS/MS spectrum of mass of interest *m*/*z* 460.9310 in the sample acquired using DDA mode (CE -60 eV).

Table S1. Chromatographic conditions of the analysis

Time (min.)	Flow rate (mL/min.)	Mobile phase %		Valve position
		A	B	
0.00	0.4	90	10	Waste
1.00	0.4	90	10	Waste
1.50	0.4	50	50	Mass spec.
8.50	0.4	1	99	Mass spec.
13.50	0.4	1	99	Mass spec.
14.00	0.4	90	10	Waste
23.00	0.4	90	10	Waste

Table S2. SWATH window parameters

Precursor ion start mass (Da)	Precursor ion end mass (Da)	DP (V)	DP spread (V)	CE (V)	CE spread (V)	Time bin to sum
100.0000	150.0000	-20	0	-35	30	4
149.0000	190.0000	-20	0	-35	30	4
199.0000	250.0000	-20	0	-35	30	4
249.0000	300.0000	-20	0	-35	30	4
299.0000	350.0000	-20	0	-35	30	4
349.0000	400.0000	-20	0	-35	30	4
399.0000	450.0000	-20	0	-35	30	4
449.0000	500.0000	-20	0	-35	30	4
499.0000	550.0000	-20	0	-35	30	4
549.0000	650.0000	-20	0	-35	30	4
649.0000	800.0000	-20	0	-35	30	4
799.0000	1200.0000	-20	0	-35	30	4

Table S3. Mass labelled standards spiked to monitor the instrument and method performance

Native Analyte	Abbreviation	Mass labelled Internal Standard
Perfluorobutanoic Acid	PFBA	$^{13}C_4$-PFBA
Perfluoropentanoic Acid	PFPeA	$^{13}C_3$ PFPeA
Perfluorohexanoic Acid	PFHxA	$^{13}C_2$-PFHxA
Perfluoroheptanoic Acid	PFHpA	$^{13}C_4$-PFHpA
Perfluorooctanoic Acid	PFOA	$^{13}C_4$-PFOA
Perfluorononanoic Acid	PFNA	$^{13}C_5$-PFNA
Perfluorodecanoic Acid	PFDA	$^{13}C_2$-PFDA
Perfluoroundecanoic Acid	PFUnDA	$^{13}C_2$PFUdA
Perfluorododecanoic Acid	PFDoDA	$^{13}C_2$PFDoA
Perfluorotridecanoic Acid	PFTrDA	$^{13}C_2$PFDoA
Perfluorotetradecanoic Acid	PFTeDA	$^{13}C_2$PFTeDA
Perfluorohexadecanoic Acid	PFHxDA	$^{13}C_2$PFHxDA
Perfluorooctadecanoic Acid	PFODA	
Perfluorobutanesulphonate	PFBS	$^{13}C_3$ PFBS
Perfluoropentanesulphonate	PFPeS	$^{13}C_3$ PFBS
Perfluorohexanesulphonate	PFHxS	$^{18}O_2$-PFHxS
Perfluoroheptanesulphonate	PFHps	
Perfluorooctanesulfonate	PFOS	$^{13}C_4$-PFOS
Perfluorononanesulfonate	PFNS	
Perfluordecanesulphonate	PFDS	
Perfluordodecanesulphonate	PFDoDS	
Fluorooctane sulfonamide	FOSA	$^{13}C_8$-FOSA
N-methyl fluorooctanesulfonamidoacetic acid	N-Me FOSA	D_3-N-Me FOSA
N-ethyl fluorooctanesulfonamidoacetic acid	N-Et FOSA	D_5-N-Et FOSA
Perfluorooctane sulfonamido acetic acid	FOSAA	
N-ethyl perfluorooctanesulfonamidoacetic acid	N-Et FOSAA	D_5-N-Et FOSAA
N-methyl perfluorooctanesulfonamidoacetic acid	N-Me FOSAA	D_3-N-Me FOSAA
N-ethyl perfluorooctane sulfonamidoethanol	N-Et FOSE	D_9-N-Et FOSE
N-methyl perfluorooctane sulfonamidoethanol	N-Me FOSE	D_7-N-Me FOSE
Perfluoro-4-ethylcyclohexanesulfonates	PFECHS	
10:2 Fluorotelomer sulfonate	10:2 FTS	$^{13}C_2$-8:2 FTS
8:2 Fluorotelomer sulfonate	8:2 FTS	
6:2 Fluorotelomer sulfonate	6:2 FTS	$^{13}C_2$6:2 FTS
4:2 Fluorotelomer sulfonate	4:2 FTS	$^{13}C_2$ 4:2 FTS
6:2 Fluorotelomer phosphate ester	6:2 PAP	$^{13}C_2$-6:2 PAP
8:2 Fluorotelomer phosphate ester	8:2 PAP	$^{13}C_2$-8:2 PAP
Perfluorooctane sulfonamide phosphate ester	SAmPAP	
6:2 Fluorotelomer phosphate diester	6:2 DiPAP	$^{13}C_4$-6:2 DiPAP
8:2 Fluorotelomer phosphate diester	8:2 DiPAP	$^{13}C_4$-8:2 DiPAP
6:2 8:2 Fluorotelomer phosphate diester	6:2 8:2 DiPAP	

Table S4. Feature finding parameters used for initial feature list generation

Workflow	Non-targeted Screening
Signal-to-noise algorithm	Relative Noise
Integration algorithm	MQ4
**Integration**	
XIC width / Da	0.02 Da
RT half window	30 sec
Minimum peak width	3 points
Minimum peak height	2000 cps
S/N integration threshold	3
Gaussian smooth width	0.8 points
Noise Percentage / %	20%
Baseline subtract window	0.5 min
Peak splitting	2 points
**Library search**	
Library search algorithm	Candidate Search
Results sorted by	Fit
Precursor mass tolerance	0.1 Da
Fragment mass tolerance	0.5 Da

Table S5: The list of features remains unidentified. Mass-to-charge ratio (*m*/*z*), retention time (RT), Kendrick Mass defect (KMD), identification (ID) confidence and the corresponding MS/MS (MS2) fragments are given for each feature. Green squares indicate the presence of the feature in whole blood (B) and serum (S) samples.

B	S	Feature *m/z*	RT	KMD	ID confidence	MS_2 fragments detected
green	grey	177.0562	2.12	0.068	5b	79.9573, 107.0499
green	grey	188.9862	1.54	0.002	5b	79.9573, 91.0194, 108.0213, 109.0291, 188.9860
grey	green	195.0689	2.96	-0.081	5b	79.9775, 96.9600
green	grey	201.0226	2.43	-0.035	5b	79.9576, 106.0427, 121.0659, 146.0615, 167.1092, 201.0228
grey	green	204.0665	2.24	-0.080	5b	72.9930, 79.9575, 109.0248, 116.0505, 128.0500, 130.0661, 142.0657, 158.0610, 186.0560, 204.0655
green	grey	204.9464	3.19	0.040	4	61.0078, 89.0026, 124.9800, 160.9568, 204.9460
green	green	213.0225	2.65	-0.036	5b	76.97, 89.0237, 133.0655, 199.1136
green	grey	215.0379	2.87	-0.052	5b	79.9576, 106.0426, 135.0813, 215.0385
grey	green	219.9302	4.68	0.056	5b	Very low intensity
green	grey	220.9414	3.28	0.044	5b	77.0030, 112.9801, 140.9742, 176.9514, 202.9310, 220.9420
green	grey	226.0179	2.23	-0.032	5b	79.9592, 131.0381, 146.0612, 226.0180
green	grey	229.0536	3.24	-0.068	5b	78.9593, 112.9807, 149.0970, 209.0952
grey	green	249.0227	3.91	-0.039	5b	Very low intensity
grey	green	249.0764	3.81	-0.092	5b	79.9572, 96.9601, 132.0040, 190.0100, 209.0854, 233.9983
grey	green	256.9085	2.75	0.075	5b	176.9512, 197.0640
grey	green	260.9863	3.83	-0.003	5b	79.9576, 145.0298, 189.0923, 216.9981
green	green	266.9644	2.13	0.019	4	79.9573, 80.9657, 107.0500, 187.0065
green	grey	270.0806	2.43	-0.098	5b	79.9571, 106.9809, 124.0073, 162.0916, 270.0799
green	green	270.9737	2.62	0.009	5b	79.9571, 135.0266, 159.0449, 191.0615, 270.9733
green	green	277.0213	2.76	-0.039	5b	79.9569, 106.0424, 133.0658, 185.9992, 213.0220, 261.9984
green	grey	281.0665	2.42	-0.084	5b	79.9579, 171.1020, 201.0231, 245.0451, 270.0809
green	grey	293.0491	2.83	-0.068	5b	79.9569, 106.0424, 185.9987, 213.0924
grey	green	296.9733	4.73	0.008	4	79.9563, 96.9592, 115.0744, 221.1902, 255.1596, 283.1917

		308.9437	3.52	0.037	**5a**	79.9569, 80.9651, 228.9864
		309.0436	2.34	-0.063	**5b**	63.9623, 80.9654, 90.9859, 134.9752, 178.9653, 229.0875, 309.0441
		379.0106	2.07	-0.035	**5b**	79.9560, 123.0450, 153.0184, 203.0012
		382.9413	4.66	0.034	**5a**	82.9606, 118.9926, 168.9882, 318.9803
		397.0037	2.11	-0.029	**5b**	79.9572, 107.0499, 187.0064
		422.9480	4.86	0.025	**5b**	Very low intensity
		435.0391	3.65	-0.067	**5b**	152.9953, 202.9992, 218.0216, 233.0457, 337.0710, 355.0843, 435.0403
		453.0662	2.89	-0.095	**5b**	79.9572, 135.0810, 215.0378, 453.0659
		480.9399	5.48	0.029	**5b**	79.9570, 80.9645, 98.9555, 118.9919, 460.9341
		611.8569	5.96	0.104	**5b**	Very low intensity
		666.8565	3.89	0.101	**5b**	Very low intensity

REFERENCES

(1) Charbonnet, J. A.; McDonough, C. A.; Xiao, F.; Schwichtenberg, T.; Cao, D. P.; Kaserzon, S.; Thomas, K. V.; Dewapriya, P.; Place, B. J.; Schymanski, E. L.; et al. Communicating Confidence of Per- and Polyfluoroalkyl Substance Identification via High-Resolution Mass Spectrometry. *Environ Sci Tech Let* **2022**, *9* (6), 473-481.
